# Efficacy and safety of Piclidenoson in the treatment of plaque psoriasis: a systematic review and meta-analysis of randomized controlled trials

**DOI:** 10.1007/s00403-024-03506-y

**Published:** 2024-11-16

**Authors:** Muhammad Zain Ul Haq, Saad Ashraf, Ayesha Shaukat, Laveeza Fatima, Muhammad Shahmeer Ullah Shah, Muhammad Ahsan Ansari, Muhammad Nabeel Saddique, Gharira Batool, Javed Iqbal

**Affiliations:** 1 Department of Medicine, Dow University of Health Sciences, Karachi, Pakistan; 2 Allama Iqbal Medical College, Lahore, Pakistan; 3 King Edward Medical University, Lahore, 54000 Pakistan; 4 Liaquat National Hospital and Medical College (LNHMC), Karachi, Pakistan; 5 Nursing Department, Communicable Disease Center, Hamad Medical Corporation, P.0 Box 3050, Doha, Qatar

**Keywords:** Piclidenoson, Psoriasis, Plaque psoriasis, A3 adenosine receptor, A3AR agonist, Meta-analysis

## Abstract

Psoriasis, an autoimmune inflammatory disease characterized by hyperproliferation of keratinocytes, affects 0.2–4.8% of the global population. The disease manifests primarily as plaque psoriasis, causing chronic physical and psychological burdens. Although numerous treatments exist, there is ongoing exploration for novel therapies due to concerns about the toxicity, efficacy, and costs of current options. An A3 adenosine receptor (A3AR) agonist called Piclidenoson has been shown to reduce inflammation and could be used to treat moderate to severe psoriasis. This meta-analysis evaluates the safety and efficacy of Piclidenoson in psoriasis treatment. This meta-analysis was conducted accordance with the PRISMA guidelines and has been registered on PROSPERO (CRD42024566459). A comprehensive search on Cochrane CENTRAL, PubMed/MEDLINE, and Google Scholar was conducted up to July 2024. Included studies were randomized controlled trials (RCTs) involving Piclidenoson. Efficacy outcomes included PASI 75 and PGA 0 or 1, while safety outcomes included adverse events. Pooled outcomes were presented as odds ratio (OR) with 95% confidence intervals (CI). Statistical analysis employed the Mantel-Haenszel random-effects model, and heterogeneity was assessed using the I^2^ and X^2^index. Three RCTs with 574 patients (313 Piclidenoson, 261 placebo) met the inclusion criteria. The pooled analysis showed no significant difference in achieving PASI 75 between Piclidenoson and placebo (OR: 1.62, 95% CI 0.70–3.75, *P* = 0.26, I^2^ = 12%). However, Piclidenoson significantly improved PGA scores (OR: 2.74, 95% CI 1.22–6.16, *P* = 0.01, I²=0%). Safety assessment revealed no significant differences in adverse events, including nervous system, gastrointestinal, musculoskeletal, renal, and infections, compared to placebo. Piclidenoson demonstrates a significant improvement in PGA scores and a favorable safety profile, suggesting it could be a valuable addition to plaque-like psoriasis treatment. Further research with larger, longer-term RCTs is needed to confirm its efficacy and optimize clinical use.

**Supplementary Information:**

The online version contains supplementary material available at 10.1007/s00403-024-03506-y.

## Introduction

Psoriasis is an autoimmune inflammatory disease characterized by cutaneous plaques resulting from the hyperproliferation of keratinocytes, likely due to their resistance to apoptosis. Psoriasis affects between 0.2% and 4.8% of the global population, with about 2% of the U.S. population impacted [1, 2]. The average age of onset for the initial presentation of psoriasis typically ranges from 15 to 20 years, with a second peak occurring between 55 and 60 years [1]. While the exact etiology remains unknown, psoriasis is considered an autoimmune disease mediated by T lymphocytes, with a significant association of HLA antigens in many patients, especially across various racial and ethnic groups, indicating a genetic predisposition [3]. The most common form, plaque psoriasis, accounts for 85–90% of cases and is characterized by erythematous, asymmetric plaques with thick scales commonly found on the extensor surfaces, trunk, and scalp [4]. While there is no cure for psoriasis, the disease typically waxes and wanes with flare-ups, contributing to a chronic physical and psychological burden [3].

A wide range of treatments is available for psoriasis, each suited to varying degrees of severity. Mild to moderate cases often benefit from topical therapies like emollients, moisturizers, coal tar, dithranol, corticosteroids, vitamin D analogs, and retinoids [3]. In contrast, severe psoriasis typically requires systemic treatments such as oral immunomodulators like methotrexate and ciclosporin or biologics—monoclonal IgG molecules (excluding etanercept) that target specific cytokines or receptors [5]. Despite the approval of biological therapies targeting these cytokines over the last few decades, concerns about their potential toxicity, reduced efficacy over time, disease recurrence, and high costs have prompted ongoing exploration into novel strategies for managing psoriasis [5].

The A3 adenosine receptor (A3AR), a Gi protein-coupled cell surface receptor, is crucial role in mediating anti-inflammatory responses when activated by selective agonists [6, 7]. It is highly overexpressed in inflammatory tissues and peripheral blood mononuclear cells of individuals with psoriasis, likely due to the upregulation of transcription factors such as nuclear factor kappa B (NF-κB) and cyclic adenosine monophosphate (cAMP) response element-binding protein (CREB) in the A3AR gene promoter [8]. Piclidenoson (CF101), a highly selective and orally bioavailable A3AR agonist, leverages these pathways to induce anti-inflammatory effects by suppressing NF-κB signaling. This mechanism leads to the downregulation of pro-inflammatory cytokines and chemokines such as interleukin-1 (IL-1), interleukin-8 (IL-8), tumor necrosis factor-alpha (TNF-α), and macrophage inflammatory protein-1 alpha (MIP-1α). Moreover, Piclidenoson inhibits the proliferation of specific auto-reactive T cells and promotes apoptosis in inflammatory cells [9–11].

Currently, there are no meta-analyses or systematic reviews specifically evaluating the efficacy and safety of Piclidenoson in treating psoriasis. We conducted a meta-analysis to address the gap in the literature by evaluating the safety and efficacy of Piclidenoson as a treatment for moderate-to-severe psoriasis.

## Methods

This meta-analysis was reported in accordance with the Preferred Reporting Items for Systematic Reviews and Meta-Analyses (PRISMA) guidelines [12]. The protocol of this meta-analysis was registered in International Prospective Register of Systematic Reviews (PROSPERO) with the reference number CRD42024572306.

### Data sources and search strategy

A comprehensive search was conducted using the Cochrane CENTRAL, PubMed/MEDLINE, and Google Scholar databases from inception to July 2024. The reference lists of the retrieved articles and prior meta-analyses were also screened for any relevant articles. The search strategy imposed no restrictions on publication status or language. The search terms included relevant PubMed MeSH terms and related keywords, such as (Psoriasis vulgaris OR Chronic plaque psoriasis OR Plaque psoriasis OR Psoriasis) AND (Piclidenoson OR CF101 OR A3AR agonist OR A3 adenosine receptor). The detailed search strategy is provided in (Supplementary Table 1).

### Study selection and eligibility criteria

All articles retrieved from the systematic search were imported into EndNote reference library, version X8.1 (Clarivate Analytics), where duplicates were subsequently removed. Two authors (ZH and SS) independently reviewed and selected studies, with any disagreements resolved by a third author (SA). Selected studies were retrieved for full-text review to confirm their relevance. The Population, Intervention, Control and Outcomes(PICOS) format for systematic reviews was used to define the inclusion criteria, with P beings trials assessing patients with Plaque Psoriasis, I being Piclidenoson, C being placebo and O being safety and efficacy outcomes.

### Data extraction and endpoint definitions

Two authors (Z.H AND S.S) conducted independent evaluations of the data and supplementary materials, on a pre-piloted Microsoft Excel sheet, with discrepancies resolved through consultation with a third author (SA). The following data were extracted from included studies: study labels, year of publication, study design, and patients’ baseline characteristics, weight, BMI, and duration of disease, as well as outcomes related to the efficacy and safety profile of the drug. The efficacy endpoints included achieving ≥ 75% improvement in PASI 75 and PGA 0 or 1 and the safety endpoints included adverse events.

### Quality assessment

The quality of included Randomized Control trials(RCTs) was assessed by two authors (ZH AND SA) using the Cochrane risk of bias tool 2.0 (RoB2.0) [13]. This tool evaluates the risk of bias related to patient selection, study performance, outcome detection, data attrition, study findings reporting, and other types of bias. The judgments can be ‘Low’ or ‘High’ risk of bias or indicate ‘Some concerns’.

### Data synthesis

Two authors (SA and SS.) conducted the data synthesis for this study using Review Manager (RevMan version 5.4; Copenhagen: The Nordic Cochrane Centre, The Cochrane Collaboration, 2014). We employed the Mantel-Haenszel random-effects model to synthesize the results across all included studies and presented them in forest plots. Statistical significance was defined as p-value < 0.05.Standardized Mean Differences (SMDs) for continuous outcomes and Odds Ratio (OR) for binary outcomes with their 95% confidence interval (CI) were calculated. Statistical heterogeneity was assessed using the Higgins I ^2^ index. (I^2^ < 50%=low, I^2^ 50–75%=moderate and I^2^ > 75%=high heterogeneity). The funnel plots to assess publication bias were not employed as limited number of studies as per recommendations of Cochrane Collaboration.

## Results

### Study selection process

**Fig. 1:**
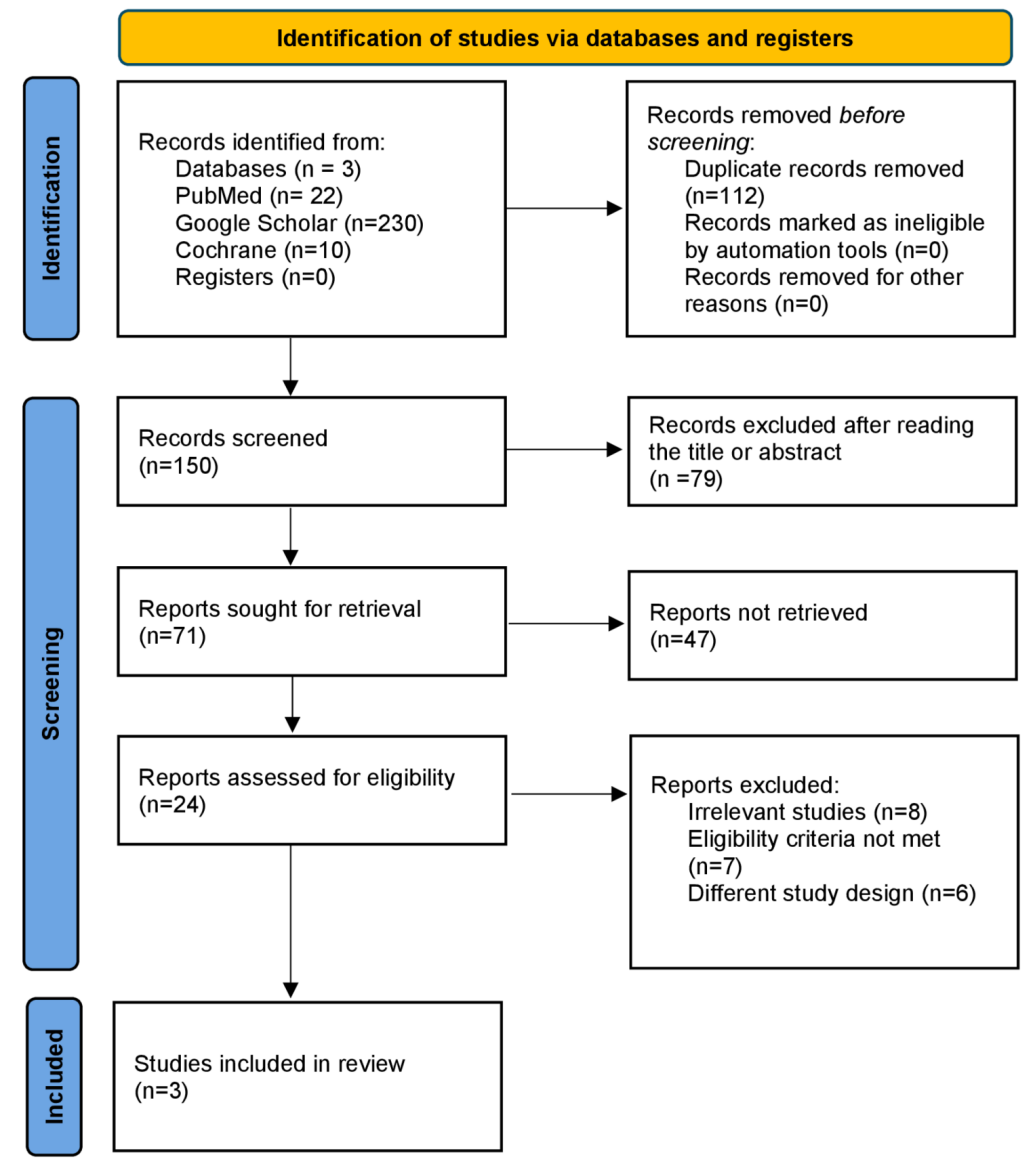
PRISMA flow diagram of the number of studies screened and included in the systematic review of the number of studies screened and included in the systematic review

The preliminary literature search yielded 262 results, which were screened for relevance based on titles, abstracts, and full-text reviews. Ultimately, 3 studies met the pre-defined inclusion criteria and were included in this systematic review [10, 14, 15]. The search and screening process is illustrated in the PRISMA flow chart (Fig. 1). In total, 574 patients were included in 3 randomized controlled trials (RCTs), with 313 patients in the intervention group and 261 in the placebo group. The route of piclidenoson administration in all 3 studies was oral. The studies administered the drug at doses of either 1, 2, 3 or 4 mg twice daily (BID) over a duration of 12–32 weeks. The basic characteristics and outcomes of the included studies are detailed in (Tables 1and 2).

**Table 1:** Baseline characteristics

Study	No. of patients		Male, *n* (%)		Age, median (range), years		Weight, median (range), Kg		BMI, median (range), Kg/m2		Race, *n* (%)		Ethnicity, *n* (%)		Duration of disease, median (range), years		PASI mean (SD)	
	Piclidenoson	Placebo	Piclidenoson	Placebo	Piclidenoson	Placebo	Piclidenoson	Placebo	Piclidenoson	Placebo	Piclidenoson	Placebo	Piclidenoson	Placebo	Piclidenoson	Placebo	Piclidenoson	Placebo
Michael David et al., 2016	145	148	91 (62.8%)	95 (64.2%)	48.3 ± 13.57	50.0 ± 13.96	83.4 ± 17.67	83.3 ± 17.42	28.33 ± 5.143	28.52 ± 5.156	White/Caucasian: 144 (99.3%)	White/Caucasian: 146 (98.6%)	N/A	N/A	11.8 ± 11.91	11.3 ± 10.02	21.5 ± 9.51	21.5 ± 10.11
K. A. Papp et al.,2024	151	94	85 (56.3%)	55 (58.5%)	50 (20–78) median (range), years	47 (19–77) median (range), years	82 (45–135) median (range), kg	82 (41–138) median (range), kg	28.3 (19.0–51.0) median (range), kg/m2	27.5 (16.2–43.1) median (range), kg/m2	white: 150 (99.3%)	white: 94 (100.0%)	Hispanics: 1 (0.7%), Non-Hispanics: 150 (99.3%)	Hispanics: 0 (0.0%), Non-Hispanics: 94 (100.0%)	12 (0–61), median (range), years	11.5 (1–45), median (range), years	N/A	N/A
Michael David et al., 2012	17	19	15 (88.2%)	14 (73.6%)	48.4 ± 2.5	51.2 ± 2.4	83.7 ± 2.7	84.2 ± 2.6	N/A	N/A	N/A	N/A	N/A	N/A	19.4 ± 2.	23.5 ± 2.5	22.9 ± 2.3	21.5 ± 10.11

*BMI = Body mass index; SD = Standard deviation;

**Table 2:** Efficacy and safety outcomes

Study	Efficacy outcome				Adverse effects													
	Achieved PASI 75 at 12 weeks		Achieved PGA 0 or 1		At least one adverse event		Musculoskeletal and connective tissue disorders		Nervous system disorders		Renal and urinary disorders		Gastrointestinal		Infections and infestations		Severe adverse event	
	Piclidenoson	Placebo	Piclidenoson	Placebo	Piclidenoson	Placebo	Piclidenoson	Placebo	Piclidenoson	Placebox	Piclidenoson	Placebo	Piclidenoson	Placebo	Piclidenoson	Placebo	Piclidenoson	Placebo
Michael David et al., 2016	12 / 141 (8.5%)	10 / 144 (6.9%)	9 / 141 (6.4%)	5 / 144 (3.5%)	37 / 145 (25.5%)	29 / 148 (19.6%)	5 / 145 (3.5%)	2 / 148 (1.4%)	6 / 145 (4.1%)	0 / 148 (0%)	5 / 145 (3.4%)	4 / 148 (2.7%)	8 / 145 (5.5%)	3 / 148 (2.0%)	10 / 145 (6.9%)	13 / 148 (8.8%)	4 / 145 (2.7%)	1 / 148 (0.6%)
K. A. Papp et al., 2024	10 / 127 (7.8%)	2 / 78 (2.5%)	15 / 127 (11.8%)	3 / 78 (3.8%)	50 / 179 (27.9%)	32 / 94 (34.0%)	2 / 179 (1.1%)	2 / 94 (2.1%)	3 / 179 (1.6%)	4 / 94 (4.2%)	1 / 179 (0.5%)	2 / 94 (2.1%)	5 / 179 (2.7%)	1 / 94 (1.0%)	20 / 179 (11.1&)	15 / 94 (15.9%)	1 / 179 (0.5%)	0 / 94 (0%)
Michael David et al., 2012	N/A	N/A	4 / 17 (23.5%)	0 / 19 (0%)	3 / 17 (17.6%)	4 / 19 (21.0%)	0 / 17 (0%)	0 / 19 (0%)	0 / 17 (0%)	0 / 19 (0%)	0 / 17 (0%)	0 / 19 (0%)	0 / 17 (0%)	0 / 19 (0%)	3 / 17 (17.6%)	0 / 19 (0%)	0 / 17 (0%)	1 / 19 (5.2%)

*PASI: Psoriasis Area and Severity Index; PGA: Physician Global Assessment

### Risk of bias assessment

The evaluation revealed all three randomized controlled trials were classified as “low-risk.” The quality assessment of the included studies is given in (Supplementary Figure S1). The studies included in this review had well-defined research objectives and purposes.

### Efficacy outcomes

#### The psoriasis area and severity index (PASI) 75

**Fig. 2:**
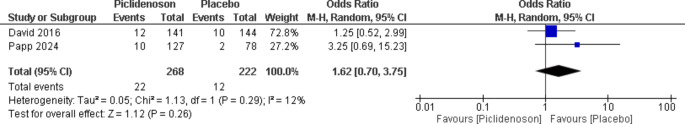
Forest plot PASI 75

Two studies reported achieving PASI 75 [10, 15]. According to the pooled analysis shown in (Fig. 2), there was no significant difference between the Piclidenoson and control groups in achieving PASI 75 [OR 1.62; 95% CI 0.70–3.75; *P* = 0.26, I^2^ = 12%].

#### Physician global assessment (PGA) score of 0 or 1

**Fig. 3:**
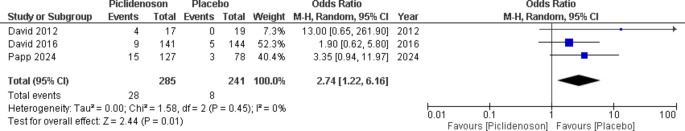
Forest Plot PGA 0 or 1

Three studies reported achieving a PGA score of 0 or 1 [10, 14, 15]. According to the pooled analysis shown in (Fig. 3), there was a significant difference between the Piclidenoson and control groups achieving a PGA score of 0 or 1 [OR 2.74; 95% CI 1.22–6.16; *P* = 0.01, I^2^ = 0%].

### Safety outcomes

#### Nervous system disorders

**Fig. 4:**
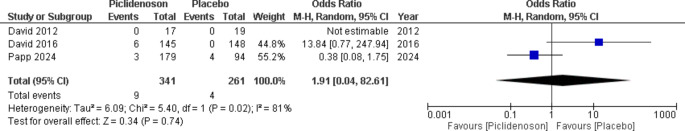
Forest plot nervous system disorders

Three studies reported nervous system disorders in the patients [10, 14, 15]. According to the pooled analysis shown in (Fig. 4), there was no significant difference between the Piclidenoson and control groups in terms of experiencing nervous system disorder [OR1.91; 95% CI 0.04–82.61; *P* = 0.74; I^2^ = 81%].)

#### GIT disorders

**Fig. 5:**
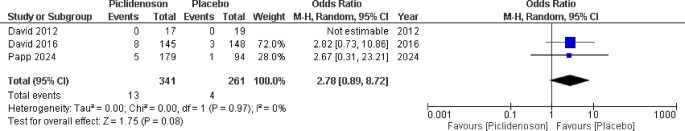
Forest Plot GIT Disorders

Three studies reported gastrointestinal tract disorders in the patients [10, 14, 15]According to the pooled analysis shown in (Fig. 5), there was no significant difference between the Piclidenoson and control groups in terms of experiencing GIT disorders [OR 2.78; 95% CI 0.89–8.72; *P* = 0.08; I^2^ = 0%].

#### Musculoskeletal and connective tissue disorders

**Fig. 6:**
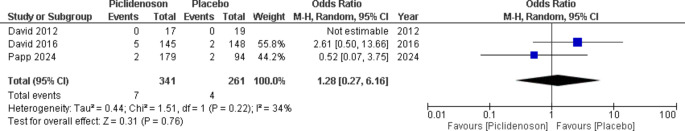
Forest plot musculoskeletal and connective tissue disorders

Three studies reported musculoskeletal (MSK) and connective tissue (CT) disorders in the patients [10, 14, 15]. The pooled analysis shown in Fig. 6 demonstrated no significant difference between the Piclidenoson and control groups in terms of experiencing MSK and CT disorders [OR 1.28; 95% CI 0.27–6.16; *P* = 0.76; I^2^ = 34].

#### Renal and urinary disorders

**Fig. 7:**
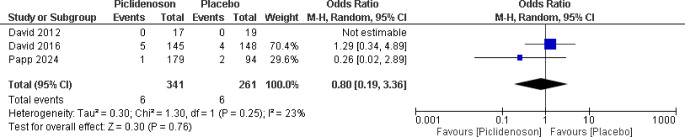
Forest plot renal and urinary disorders

Three studies reported musculoskeletal, renal, and urinary disorders in the patients [10, 14, 15]. According to the pooled analysis shown in Fig. 7, there was no significant difference between the Piclidenoson and control groups in terms of experiencing renal and urinary disorders [OR 0.80; 95% CI 0.19–3.36; *P* = 0.25, I^2^ = 23%].

#### Infections and infestations

**Fig. 8:**
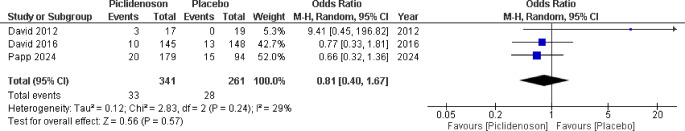
Forest plot infection and infestation

Three studies reported infections and infestations in the patients [10, 14, 15].The pooled analysis shown in Fig. 8, demonstrated no significant difference between the Piclidenoson and control groups in terms of experiencing renal and urinary disorders[OR 0.81; 95% CI 0.40–1.67; *P* = 0.57; I² =29%].

#### At least one adverse events (AE)

**Fig. 9:**
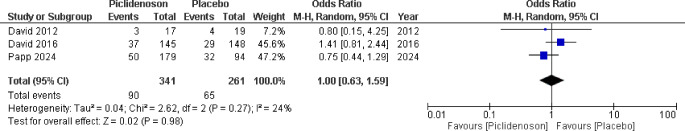
Forest plot at least one adverse event

Three studies reported at least one adverse event (AE) [10, 14, 15]. The pooled analysis shown in Fig. 9, displayed that there was no significant difference between the Piclidenoson and control groups in terms of experiencing at least one AE [OR 1.00 95% CI 0.63–1.59; *P* = 0.98; I² 24%].

#### Severe adverse event (SAE)

**Fig. 10:**
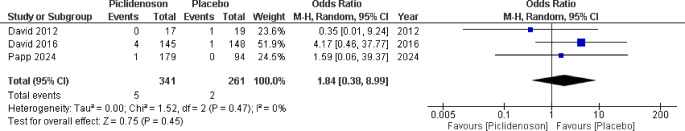
Severe adverse event

Severe adverse events (SAE) rates were reported in three studies [10, 14, 15] According to the pooled analysis shown in Fig. 10, there was no significant difference between the Piclidenoson and control groups in terms of experiencing severe adverse events [OR 1.84; 95% CI:0.38–8.99; *p* = 0.45; I²= 0%].

## Discussion

To the best of our knowledge, this is the first meta-analysis to evaluate the efficacy and safety of Piclidenoson in treating psoriasis. Our systematic review and meta-analysis revealed that despite not achieving a statistically significant improvement in PASI 75 scores, Piclidenoson demonstrated a notable benefit in achieving Physician’s Global Assessment (PGA) scores of 0 or 1. Similarly, common adverse effects were also comparable between Piclidenoson and placebo.

Regarding efficacy, the two studies [10, 15] included in our analysis that reported on PASI 75 achievement did not show a significant difference between the Piclidenoson and placebo groups. PASI 75 refers to a 75% reduction in the Psoriasis Area and Severity Index score, which quantifies disease severity based on the extent of skin involvement, redness, thickness, and scaling [16]. This suggests that while Piclidenoson may not substantially reduce psoriasis severity by 75% as measured by PASI 75, it does positively impact on the overall clinical appearance of the disease. This discrepancy could be due to several factors, including variations in patient demographics, the specific dosage regimens used in the studies, and the duration of treatment. The lack of a significant difference in PASI 75 scores highlights the need for further research to determine the optimal conditions under which Piclidenoson might be most effective.

Conversely, the analysis of PGA scores, reported in three studies [10, 14, 15], indicated a significant improvement with Piclidenoson compared to placebo. The PGA evaluates the overall severity of psoriasis, focusing on the extent and intensity of plaques, and is scored on a scale where 0 or 1 indicates ‘clear’ or ‘almost clear,’ representing a significant improvement in clinical appearance [16]. Patients treated with Piclidenoson were likelier to achieve a PGA score of 0 or 1, suggesting a clear clinical improvement. This outcome is particularly relevant as PGA scores provide a comprehensive assessment of disease severity, considering the extent and intensity of psoriasis. Achieving a PGA score of 0 or 1 implies that patients experienced minimal to no visible signs of psoriasis, which is a crucial indicator of treatment success from both a clinical and patient perspective.

The PASI 75 score, a widely accepted clinical endpoint in psoriasis studies, is critical in evaluating treatment efficacy. PASI is a more detailed and well-validated index that quantifies psoriasis severity based on body surface area (BSA) involvement, plaque redness, thickness, and scaling, making it ideal for comprehensive clinical trials [16]. However, PASI has limitations, particularly its sensitivity to detect changes in patients with milder psoriasis or localized disease. In contrast, the PGA offers a simpler, clinician-friendly evaluation of overall disease severity. It relies on visual assessment and considers factors such as plaque elevation, erythema, and scaling across all affected areas, without requiring BSA calculations [16]. The ease of its use makes the PGA highly relevant in both clinical trials and routine practice. Moreover, a PGA score of 0 or 1 (clear or almost clear) indicates that the patient experiences minimal to no visible signs of disease, which can be a critical factor in measuring patient satisfaction and quality of life. The significant improvement in PGA scores observed in this analysis further supports its clinical relevance and potential as a complementary or alternative tool to PASI in evaluating treatment efficacy. Given these points, the PGA should be regarded as a valid and meaningful endpoint in this study, particularly since it simplifies assessment without compromising accuracy.

Safety outcomes were also thoroughly examined, with multiple domains analyzed to assess the incidence of adverse events (AEs). Commonly reported adverse events, such as nervous system disorders, included symptoms like headaches and dizziness. These events were observed at comparable rates between the Piclidenoson and placebo groups, indicating that Piclidenoson does not appear to increase the risk of these issues. Similarly, GIT disturbances, including nausea and digestive discomfort, were reported, but again, there was no indication that Piclidenoson posed a higher risk compared to placebo. Musculoskeletal and connective tissue disorders, which can sometimes arise with psoriasis treatments, were similar across both groups, suggesting that Piclidenoson does not contribute to an elevated risk of joint or muscle issues. Additionally, no marked increase in renal or urinary disorders was observed, and the incidence of infections was similar in both treatment groups. Given the chronic nature of psoriasis, the ability to manage the disease with a treatment that does not introduce significant long-term safety concerns is a considerable advantage. The similarity in AE rates further supports the idea that Piclidenoson can be considered safe for patients with plaque-like psoriasis, offering an alternative for those with contraindications to other treatments.

Overall, AE rates were similar between the Piclidenoson and placebo groups, with approximately one-quarter of patients in both groups reporting at least one AE. This parity in AE rates indicates that Piclidenoson does not introduce additional safety risks compared to placebo. Similarly, the rate of severe adverse events (SAEs) did not differ significantly between the groups, further supporting the safety of Piclidenoson.

Our findings are consistent with previous studies, including the phase 3 trial by Papp et al. (2024) and the phase 2 trial by David et al. (2012) [10, 14, 15]. These studies consistently demonstrate that Piclidenoson effectively reduces psoriasis severity, achieving significant improvements in PASI and PGA scores while maintaining a favorable safety profile. The aggregation of data in our meta-analysis provides a comprehensive understanding of Piclidenoson’s efficacy and safety across diverse patient populations and clinical settings. The robust evidence from the COMFORT-1 trial, due to its larger scale and longer duration, supports the use of Piclidenoson. Our study further strengthens these findings by mitigating potential biases and enhancing the reliability of the results, thereby highlighting Piclidenoson as a viable and cost-effective treatment for moderate-to-severe psoriasis.

The current guidelines by the American Academy of Dermatology (AAD) recommend biologics and systemic treatments like methotrexate, cyclosporine, and newer biologics targeting IL-17, IL-23, and TNF-α for moderate to severe psoriasis. Biologics, particularly IL-17 and IL-23 inhibitors, such as bimekizumab, brodalumab, and risankizumab, have demonstrated significantly faster and higher rates of achieving PASI 75 and PASI 90 in clinical trials [17]. For instance, IL-17 inhibitors can achieve PASI 75 in as little as 3.4 weeks, considerably faster than many older therapies [17] ​. Moreover, these newer treatments offer robust efficacy, with some studies showing upwards of 90% efficacy in achieving PASI 75​. The slower onset and lower efficacy of Piclidenoson may limit its competitive edge in the treatment landscape, especially in patients requiring rapid relief from severe symptoms. This stark contrast in efficacy highlights the need for a more precise and cautious presentation of Piclidenoson’s potential as a therapeutic option. Nevertheless, its place in therapy, mainly when compared to established biologics, should be considered cautiously, emphasizing its limited PASI 75 efficacy and positioning it as a possible adjunct rather than a replacement for more effective treatments.

Moderate-to-severe psoriasis is typically managed with systemic non biologic therapies (like methotrexate and cyclosporin), targeted therapies (such as the oral PDE4 inhibitor apremilast), and biological therapies targeting TNF-α, IL 12/23, IL-17, or IL-23. Although biologics and oral-targeted therapies have improved treatment, they are expensive, require injections, ongoing monitoring, and pose risks of serious infections. Drug survival studies show that up to 20% of patients discontinue biologics within a year due to decreased efficacy, and apremilast has even higher discontinuation rates for the same reason. Therefore, there is still a need for a treatment that is affordable, safe, and effective. Piclidenoson, with its good safety profile and consistent efficacy, could meet this need, especially given the chronic and lifelong nature of psoriasis treatment. Its favorable safety profile makes it an attractive option for specific patient populations, especially those with contraindications to biologics or systemic agents.

Clinically, our meta-analysis suggests that Piclidenoson could be considered an alternative treatment for patients seeking a different safety profile from existing therapies. The lack of significant safety concerns supports its potential role as a safe treatment option. However, it is important to note that while Piclidenoson may improve overall clinical appearance as measured by PGA scores, it may not achieve the specific PASI 75 benchmark.

Future research should prioritize larger, multicenter RCTs with extended follow-up periods and direct comparison studies between Piclidenoson and standard treatments to better establish its efficacy and safety profile. While the included studies focus on short-term outcomes, it is crucial to recognize that psoriasis is a chronic condition, and the long-term efficacy and safety of Piclidenoson hold significant promise. Although the data from these studies demonstrate positive improvements in PGA scores, future research could greatly benefit from extending the duration of follow-up to assess whether these benefits are sustained over time. Furthermore, including a more diverse patient population in terms of demographics, disease severity, and comorbidities will be essential to ensure generalizability across different patient groups. The longer-term studies may provide further insights into its potential as a durable and well-tolerated treatment option for chronic psoriasis.

However, several limitations must be acknowledged. The primary limitation is the small sample size due to the limited number of available trials, making it difficult to draw definitive conclusions about the broader usefulness of Piclidenoson in treating psoriasis. Additionally, we couldn’t assess publication bias due to the small number of studies that could influence the findings. Furthermore, the heterogeneity in patient populations, treatment regimens, and study designs across the trials complicates direct comparisons and generalizability. Finally, the lack of direct comparisons with more commonly used biologics in clinical trials limits Piclidenoson’s relative positioning in the current therapeutic landscape.

## Conclusion

In conclusion, this meta-analysis, comprising 3 RCTs, demonstrated the efficacy of Piclidenoson in achieving significant improvements in PGA scores of 0 or 1 in patients with plaque psoriasis. However, Piclidenoson did not show a statistically significant improvement in achieving PASI 75 compared to placebo. The absence of significant AEs across various systems, including nervous, gastrointestinal, and musculoskeletal disorders, supports its potential as a well-tolerated long-term treatment option. Nevertheless, the small sample size and short-term focus of the analyzed studies underscore the need for more significant, multicenter trials with extended follow-up to confirm its sustained efficacy and safety. Future studies should focus on optimizing Piclidenoson’s dosage and treatment duration and on evaluating its long-term effectiveness and safety in comparison to more established psoriasis treatments to define its role in clinical practice better.

## Electronic supplementary material

Below is the link to the electronic supplementary material.

Supplementary Material 1

Supplementary Material 2

## Data Availability

No datasets were generated or analysed during the current study.
