# Supplementary material for: Efficacy and safety of Piclidenoson in the treatment of plaque psoriasis: a systematic review and meta-analysis of randomized controlled trials
Source: Arch Dermatol Res. 2024 Nov 16;317(1):27. doi: 10.1007/s00403-024-03506-y (PMC11568974; doi:10.1007/s00403-024-03506-y)
Supplement: Supplementary file 2 — Supplementary Material 2 [file 403_2024_3506_MOESM2_ESM.docx]

**Table S1: Search Strategy Table**

| **DATABASES** | **SEARCH STRATEGY** | | **RESULTS** |
| --- | --- | --- | --- |
| PubMed | ((("Psoriasis"[MeSH Terms] OR "Psoriasis"[All Fields] OR "psoriases"[All Fields] OR "psoriasi"[All Fields]) AND "vulgaris"[All Fields]) OR (("chronic"[All Fields] OR "chronical"[All Fields] OR "chronically"[All Fields] OR "chronicities"[All Fields] OR "chronicity"[All Fields] OR "chronicization"[All Fields] OR "chronics"[All Fields]) AND ("plaque s"[All Fields] OR "plaque, amyloid"[MeSH Terms] OR ("plaque"[All Fields] AND "amyloid"[All Fields]) OR "amyloid plaque"[All Fields] OR "plaque"[All Fields] OR "dental plaque"[MeSH Terms] OR ("dental"[All Fields] AND "plaque"[All Fields]) OR "dental plaque"[All Fields] OR "plaques"[All Fields]) AND ("Psoriasis"[MeSH Terms] OR "Psoriasis"[All Fields] OR "psoriases"[All Fields] OR "psoriasi"[All Fields])) OR (("Psoriasis"[MeSH Terms] OR "Psoriasis"[All Fields] OR "psoriases"[All Fields] OR "psoriasi"[All Fields]) AND "vulgaris"[All Fields] AND "type"[All Fields]) OR (("plaque s"[All Fields] OR "plaque, amyloid"[MeSH Terms] OR ("plaque"[All Fields] AND "amyloid"[All Fields]) OR "amyloid plaque"[All Fields] OR "plaque"[All Fields] OR "dental plaque"[MeSH Terms] OR ("dental"[All Fields] AND "plaque"[All Fields]) OR "dental plaque"[All Fields] OR "plaques"[All Fields]) AND ("Psoriasis"[MeSH Terms] OR "Psoriasis"[All Fields] OR "psoriases"[All Fields] OR "psoriasi"[All Fields])) OR (("Psoriasis"[MeSH Terms] OR "Psoriasis"[All Fields] OR "psoriases"[All Fields] OR "psoriasi"[All Fields]) AND ("chronic"[All Fields] OR "chronical"[All Fields] OR "chronically"[All Fields] OR "chronicities"[All Fields] OR "chronicity"[All Fields] OR "chronicization"[All Fields] OR "chronics"[All Fields]) AND ("stationaries"[All Fields] OR "stationary"[All Fields])) OR (("Psoriasis"[MeSH Terms] OR "Psoriasis"[All Fields] OR "psoriases"[All Fields] OR "psoriasi"[All Fields]) AND ("plaque s"[All Fields] OR "plaque, amyloid"[MeSH Terms] OR ("plaque"[All Fields] AND "amyloid"[All Fields]) OR "amyloid plaque"[All Fields] OR "plaque"[All Fields] OR "dental plaque"[MeSH Terms] OR ("dental"[All Fields] AND "plaque"[All Fields]) OR "dental plaque"[All Fields] OR "plaques"[All Fields]) AND "type"[All Fields]) OR (("Psoriasis"[MeSH Terms] OR "Psoriasis"[All Fields] OR "psoriases"[All Fields] OR "psoriasi"[All Fields]) AND "vulgaris"[All Fields]) OR "Psoriasis"[MeSH Terms]) AND ("Piclidenoson"[All Fields] OR ("cf101"[Supplementary Concept] OR "cf101"[All Fields] OR "cf101"[All Fields]) OR ("A3AR"[All Fields] AND ("agonist"[All Fields] OR "agonist s"[All Fields] OR "agonistic"[All Fields] OR "agonistically"[All Fields] OR "agonistics"[All Fields] OR "agonists"[MeSH Subheading] OR "agonists"[All Fields])) OR ("A3"[All Fields] AND ("receptors, purinergic p1"[MeSH Terms] OR ("receptors"[All Fields] AND "purinergic"[All Fields] AND "p1"[All Fields]) OR "purinergic p1 receptors"[All Fields] OR ("adenosine"[All Fields] AND "receptor"[All Fields]) OR "adenosine receptor"[All Fields]))) | | 22 |
| Cochrane | #1: ("psoriasis vulgaris"):ti,ab,kw OR (Chronic plaque psoriasis):ti,ab,kw OR (Psoriasis vulgaris type):ti,ab,kw OR (Plaque psoriasis):ti,ab,kw OR (Psoriasis):ti,ab,kw  #2: (CF101):ti,ab,kw OR (Piclidenoson):ti,ab,kw  #1 AND #2 | 10106  32  10 | |
| Google Scholar | (Psoriasis vulgaris) OR (Chronic plaque psoriasis) OR (Psoriasis vulgaris type) OR (Plaque psoriasis) OR (Psoriasis, chronic stationary) OR (Psoriasis, plaque type) OR (Psoriasis, vulgaris) OR (Psoriasis) AND (Piclidenoson) OR (CF101 OR A3AR agonist) OR (A3 adenosine receptor) | 230 | |


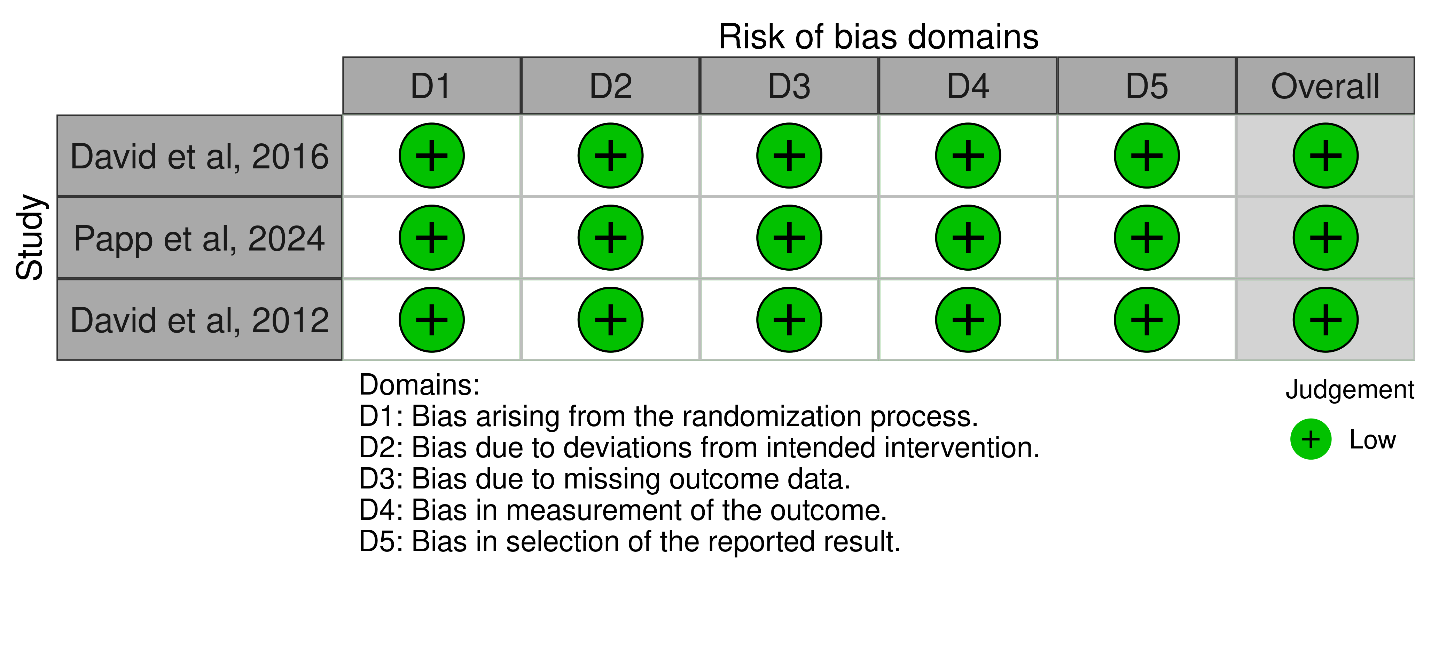


**Figure S1: ROB2 Traffic Light Plot showing the risk of bias across included studies. Colors indicate risk levels: green for low risk, yellow for some concerns, and red for high risk**


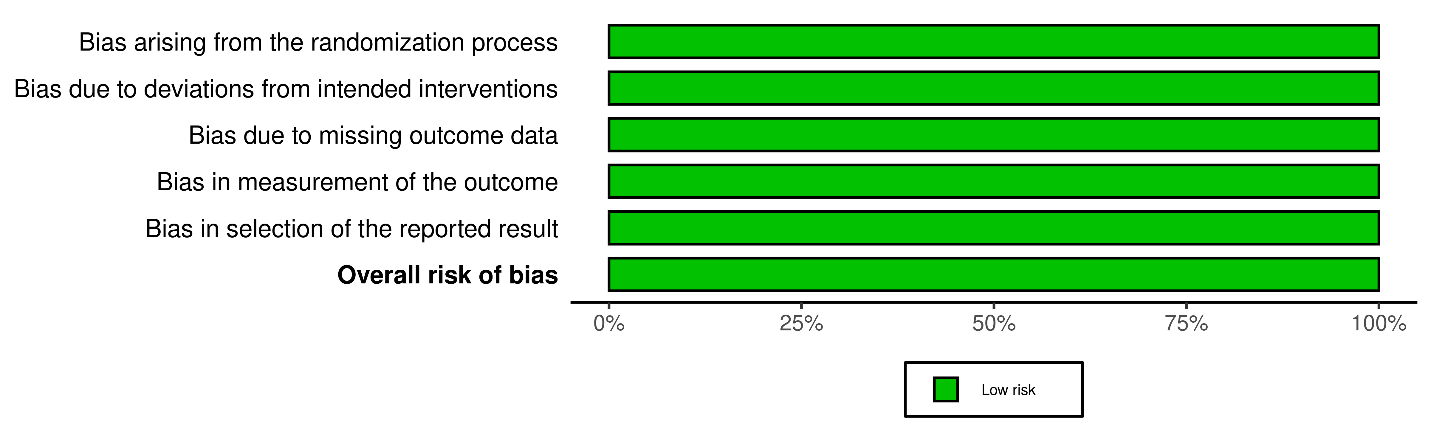


**Figure S2: Weighted bar plot of the Risk of Bias (ROB2) assessment for included studies. Green indicates low risk, yellow indicates some concerns, and red indicates high risk of bias**
